# CD4 trajectory adjusting for dropout among HIV-positive patients receiving combination antiretroviral therapy in an East African HIV care centre

**DOI:** 10.7448/IAS.17.1.18957

**Published:** 2014-08-14

**Authors:** Agnes N Kiragga, Judith J Lok, Beverly S Musick, Ronald J Bosch, Ann Mwangi, Kara K Wools-Kaloustian, Constantin T Yiannoutsos, for the East Africa IeDEA Regional Consortium

**Affiliations:** 1 Infectious Diseases Institute, Kampala, Uganda; 2 Department of Biostatistics, Harvard School of Public Health Boston, MA, USA; 3 Department of Biostatistics, Indiana University School of Medicine Indianapolis, IN, USA; 4 Center for Biostatistics in AIDS Research, Harvard School of Public Health Boston, MA, USA; 5 School of Medicine, Moi University, Eldoret, Kenya; 6 Division of Infectious Diseases, Indiana University School of Medicine Indianapolis, IN, USA; 7 Department of Biostatistics, Fairbanks School of Public Health, Indiana University Indianapolis, IN, USA

**Keywords:** HIV/AIDS, IPCW, Resource-limited setting, CD4 count, Mathematical modeling, sub-Saharan Africa

## Abstract

**Objective:**

Estimates of CD4 response to antiretroviral therapy (ART) obtained by averaging data from patients in care, overestimate population CD4 response and treatment program effectiveness because they do not consider data from patients who are deceased or not in care. We use mathematical methods to assess and adjust for this bias based on patient characteristics.

**Design:**

We examined data from 25,261 HIV-positive patients from the East Africa IeDEA Consortium.

**Methods:**

We used inverse probability of censoring weighting (IPCW) to represent patients not in care by patients in care with similar characteristics. We address two questions: What would the median CD4 be “had everyone starting ART remained on observation?” and “were everyone starting ART maintained on treatment?”

**Results:**

Routine CD4 count estimates were higher than adjusted estimates even under the best-case scenario of maintaining all patients on treatment. Two years after starting ART, differences between estimates diverged from 30 cells/µL, assuming similar mortality and treatment access among dropouts as patients in care, to over 100 cells/µL assuming 20% lower survival and 50% lower treatment access among dropouts. When considering only patients in care, the proportion of patients with CD4 above 350 cells/µL was 50% adjusted to below 30% when accounting for patients not in care. One-year mortality diverged 6–14% from the naïve estimates depending on assumptions about access to care among lost patients.

**Conclusions:**

Ignoring mortality and loss to care results in over-estimation of ART response for patients starting treatment and exaggerates the efficacy of treatment programs administering it.

## Introduction

CD4+T-cell (CD4) count is a marker for disease progression in HIV-positive patients [1, 2]. For patients on combination antiretroviral therapy (ART) in resource-limited settings, who do not have access to routine viral load testing, changes in CD4 counts constitute an important component in patient monitoring and evaluation of treatment response [3]. With rapid increases in the availability of treatment, including second-line therapies, plus new guidelines advocating the initiation of ART at higher CD4 counts [4], it is important to derive accurate estimates of the immunological impact of ART among HIV-positive patients at the program level. Decisions about the effectiveness of therapy or the success of a program, as well as policies about the optimal use of ART, are directly influenced by assessments of the immunologic response of the treated population.

Previous studies have reported robust immunological response among patients initiating ART in low- and middle-income settings [5–7]. However, these studies invariably base their findings on averaged CD4 counts among patients who are alive and still in care. Given that a low CD4 count is a predictor of both adverse clinical outcome [5, 8, 9] and loss from follow-up [9–11], patients with lower CD4 counts will be disproportionately more likely to discontinue from care or die. Patients discontinue care for a number of reasons, including illness and lack of adherence, which are strongly associated with lower CD4 counts and discontinuation from care [12]. Thus, any estimate which excludes these individuals is expected to overestimate, perhaps substantially, the response to therapy, leading to erroneous conclusions about treatment efficacy and program effectiveness [12]. In addition, exclusion of deaths from the estimation fails to incorporate patient survival, clearly the ultimate measure of program effectiveness. This likely adversely affects policy and decision-making. For these reasons, it would be desirable to carry out an analysis of CD4 response to ART, which would encompass all patients who started therapy, similar to an intent-to-treat analysis in clinical trials. Such an analysis would be helpful when, for example, we compare the effectiveness of two or more care and treatment programs or programmatic interventions. This is because comparisons based on traditional estimates of CD4 response to ART may favour programs or interventions with high dropout and death rates among their sickest patients compared to those which manage to maintain a higher proportion of their clients in care and under treatment. In the current paper, we use a CD4 measure adjusted for mortality, where we assign a CD4 count of −1 (lower than any conceivable CD4 count) to patients who have died up to their expected follow-up duration and estimate the median CD4 count (which is not affected by the actual value of this penalty factor). The interpretation of the median thus defined is the CD4 threshold such that 50% of the patients were alive with a CD4 count above this CD4 threshold.

Another analysis, which would be helpful for decision makers, involves questions of the response to therapy under some hypothetical intervention such as, for example, if it were possible to maintain on therapy, a higher percentage of patients starting ART. Such hypothetical scenarios have been routinely addressed through mathematical modelling but these procedures and results are frequently not transparent or well understood and are heavily influenced by unverified assumptions or hypotheses based on historical data external to the population under study. As we show in this paper, all these analyses can be approached through adjustments calculated from available data on actual rather than potential patients as well as strong but plausible assumptions on the outcome of patients who discontinue participation in care.

Obviously, a major obstacle in obtaining reasonable adjustments to address the bias generated by the anticipated informative dropout of the sickest among HIV-positive patients is the fact that it is very difficult to physically measure CD4 counts among patients who have discontinued care and impossible to do so among those who have died. To overcome this obvious limitation we can use methods such as inverse probability of censoring weighting (IPCW) [13]. The crux of the IPCW methodology is to use data from patients under observation to represent patients who have been lost to clinic with similar characteristics obtained up to their last visit. IPCW has been applied successfully to numerous situations. More relevant to our objectives, IPCW has been applied to the estimation of longitudinal CD4 counts and HIV RNA levels (viral load) in HIV-1 infected patients in the United States [14]. In that paper, CD4 counts and viral load measurements, which were unavailable on patients who discontinued participation in the study, were represented by their counterparts measured on similar patients still on observation.

One of the advantages of the IPCW approach is that it can be customized to address specific questions, some contrary to fact or “counterfactual,” which may not be directly measurable. Two examples of such questions relevant to our problem in the resource-limited setting are the following: “What would the long-term median CD4 count be if all patients starting therapy had remained under observation (but not necessarily continued to receive treatment)?” and “What would be the long-term median CD4 count had all patients starting therapy remained both in care and on treatment?”

In this article we address these two questions in the context of a large HIV care and treatment program in sub-Saharan Africa. The former question is relevant because it measures patient outcomes on all patients starting therapy (a kind of “intent to treat” analysis). The latter question measures the benefit of policies which increase patient retention in care and on treatment. The answers to these questions are therefore of substantial clinical and public health interest. If, in the process of addressing the first question, it is established that CD4 response to treatment is overestimated when not considering patients who dropped out or died, the estimated median CD4 counts after ART has been initiated will be adjusted downward and so will the effectiveness of programs with higher dropout and death rates among their clients. Similarly if, when addressing the second question, the adjustment of estimated median CD4 counts after ART is positive, the implication would be that there is a significant benefit resulting from interventions increasing patient retention in care and treatment. Such a conclusion (that CD4 response is significantly better were all patients retained in care and on treatment) would inform decision-making about, for example, the comparative effectiveness of efforts aimed at increasing patient retention in care and treatment versus focusing all program resources in initiating therapy on a higher number of patients.

## Methods

### Patients and visits

We included adult HIV-positive patients aged ≥15 years, receiving routine HIV care and initiating ART after 1st February 2004 in the United States Agency for International Development and the Academic Model Providing Access to Healthcare (USAID-AMPATH) partnership in Eldoret, Kenya [15], a member of the East Africa International Epidemiologic Databases to Evaluate AIDS (IeDEA) Consortium. Patients who initiated ART within nine months of database closure (14th March 2008) were excluded.

Most patients had monthly visits after the clinic visit when treatment was initiated. Data were summarized in six-month intervals centred at 0, 6, 12, 18 and 24 months post ART initiation ± 3 months. With the exception of the first interval, which included visits from ART start up to three months post treatment initiation, all subsequent intervals spanned six months of clinic visits (i.e. interval 2 spanned visits three and nine months after ART initiation, interval 3 between 9 and 15 months, etc.). All patients with at least one visit in each interval had a data record in the interval. The maximum World Health Organization (WHO) stage was selected from among all reported within an interval. The CD4 count and body weight closest to the midpoint of the interval were used in the analysis.

### Statistical methods

We estimated the CD4 count trajectory by the median CD4 count after ART initiation at six-month intervals. CD4 counts missing between consecutive intervals were interpolated by fitting linear segments between available measurements. CD4 counts missing continuously from after an interval until the end of follow-up were imputed by carrying forward the most recently available observation. CD4 counts missing at ART initiation were imputed as the mean estimate based on a normal model with predictors including WHO stage, gender and age at ART initiation. Other missing longitudinal measures, including weight and WHO stage were imputed in the same manner. Only single imputations were carried out, as it is not clear from the literature how multiple imputations are to be combined in the case of IPCW analyses. ART treatment status was only missing between visits so linear interpolation was used and interpolated values were rounded off to 0 or 1 (respectively no treatment and treatment). Thus, interpolation between an interval where treatment was being administered and one with no treatment, resulted in an imputed treatment cessation date about half way between two visits with observed treatment status. Similarly, a treatment initiation date was imputed about half way between an interval with no treatment and one with treatment.

We followed the methodology described by Lok and colleagues [14], so that for all time intervals after death, CD4 counts of patients who died were replaced by -1, a count below the lowest possible CD4 count of zero. This was done in order to provide a combined measure of program effectiveness which includes both CD4 response and mortality. The interpretation of this survival-adjusted median CD4 count is the CD4 threshold such that 50% of the patients were alive with a CD4 count above this CD4 threshold. In the Discussion section, we provide an alternative analysis where mortality is not included in the estimation of CD4 response to treatment.

We used IPCW methodology [13, 16] to address the problem of selective dropout, identical to methods applied in Lok *et al*. [14]. The interpretation of IPCW is that subjects who remained in care with the same measured characteristics represent subjects who were lost to clinic with the same characteristics as of their last clinic visit by increasing the weight of the observed subjects (who thus represent themselves plus a part of the lost patients who are similar to them based on factors observed prior to disengagement from care). IPCW, as described in Robins *et al*. [13, 16], requires that the probability of censoring only depends on patient characteristics measured while a patient is still in follow-up (that is, the data on patients who are lost are “Missing At Random” (MAR) [17]). IPCW is modelling the probability of remaining in care (i.e. coming to return clinic visits) or, as the question may be, both in care and on treatment. IPCW accomplishes this by using information on lost patients measured while the patient was still on observation. We used a pooled logistic regression model [18] to estimate the probability of remaining in care at each six-monthly interval. Factors included in the model were age, sex, year of ART initiation, WHO stage and CD4 count at ART initiation, defined as the closest available observation obtained within six months before and up to two weeks after treatment start, time-updated WHO stage and CD4 cell count and binary indicators of whether the patient was receiving therapy during the previous time interval. The time factor was included as a cubic spline in the pooled logistic-regression model. To estimate dropout rates, we assumed that all patients were present at the beginning of each interval while, when estimating mortality rates, we removed all dropouts from the denominator and, when estimating treatment discontinuation rates we removed all dropouts and deceased patients from the denominator (in effect assuming that, to discontinue treatment, a patient needed to be both alive and under observation). We used two distinct models of censoring: one model to predict remaining on observation in the first interval (visits between zero and three months from ART initiation), using baseline measurements only (that is, measurements available at the time of treatment initiation), and a second model to predict remaining on observation after three months from the start of therapy using both baseline and time-updated measurements. Baseline and time-varying CD4 counts, WHO stage and weight measurements as well as the type of clinic and demographic characteristics such as age and gender were used in the modelling of the probability of discontinuing from observation. Lagged longitudinal measurements (i.e. CD4 counts or weights measured in the previous interval) summarized the history of these measures up to the present interval. The need for a distinct model during the period immediately following ART initiation was due to the observed higher mortality and dropout rate during the first three months after initiation of antiretroviral treatment [19]. From a modelling perspective, a separate analysis is also necessary since, after the start of ART, the models calculate the probability of censoring given the entire history of past patient characteristics and treatment status while, for the first interval, the past extends only up to the point of the start of ART.

It is expected that a substantial proportion of patients lost to follow-up will have died and/or have discontinued treatment in proportions much higher than those seen among patients still on observation (not MAR). For this reason, we performed a sensitivity analysis considering varying mortality and treatment access rates among dropouts. In these analyses, treatment access rates among dropouts were estimated by multiplying the corresponding treatment access rates among patients under observation with similar characteristics by a factor of 0.2, 0.5 and 0.8. Similarly, the probability of being alive after discontinuation from care was estimated by multiplying the corresponding survival probability among patients under observation with similar characteristics by 0.2 and 0.5. These factors are consistent with reported mortality and treatment access patterns among dropouts in a variety of settings [11, 20–22] (see Discussion for more details). We then applied these factors in our estimate of the weights. Sensitivity analysis which assumes differential mortality and treatment access rates among patients lost to clinic contravenes accepted assumptions of data (MAR), inherent in the IPCW methodology. In other words, we acknowledge the possibility that patients lost to clinic may not be adequately represented by patients on observation based on measures available at the time of dropout. We have developed extensive theory about how to incorporate such assumptions into the IPCW setting (in effect dealing with a situation of data missing not at random – MNAR). An outline of the procedure used to carry out the sensitivity analysis has been included in Appendix 1. Details are described in a forthcoming methodological publication.

The previous analyses (minus the sensitivity analyses just described) address the counterfactual question “What would be the median CD4 count had everyone remained under observation and *in care*?” The implicit assumption is that patients who discontinue from observation continue or discontinue treatment and survive or die in the same rates as those observed among patients remaining in care. When access to care among the dropouts is considered through the sensitivity analyses, a slightly different counterfactual scenario is actually being tested. This scenario is expressed as follows: “What would be the median CD4 count had everyone remained on observation (but not necessarily in care or treatment)?” In other words, what would be the median CD4 count were we to have access to the CD4 counts and vital status on all patients who started therapy (but not necessarily assume that they are in care)? We delineate these nuances in the research questions in great detail in the Discussion.

To estimate the median CD4 count trajectory under the second counterfactual scenario, that is, “What would the median CD4 count be had everyone remained in care and on treatment?” we censored patients when they either discontinued follow-up or remained under observation but discontinued treatment (ART). We then applied the IPCW methodology. To estimate the weights, we fit a separate pooled logistic-regression model where discontinuation from care and treatment was the censoring event (as opposed to discontinuation from care/observation in Scenario 1). Again, two distinct models were fitted in the same manner as described above: one for the period between zero and three months from the start of therapy and a second model for the subsequent time intervals.

We generated 95% confidence intervals by sampling with replacement from the patient population and then performing the analysis on the sampled pseudo-population (bootstrap confidence intervals). One thousand such repetitions were generated and the 95% confidence interval corresponds to the 2.5th and 97.5th percentile of the generated medians or, in the case of the proportion of patients with CD4 counts > 350 cells/µL, the corresponding quantiles of the estimated proportion.

All analyses were performed with STATA version 13 (Stata Corporation, College Station, TX). All figures were generated using R 3.1.0 [23]

## Results

A total of 25,261 patients were included in the analysis; 9190 (36.4%) were male, the median age at ART initiation was 38 years (inter-quartile range [IQR] 32–44), the median CD4 cell count at the start of treatment was 116 cells/µL (53–180) (Table 1). Within the first three months after ART start, 2929 (11.6%) patients dropped out of follow-up and 1015 (4.5%) died while on follow-up. After the first interval, another 707 patients died while on follow-up and 3716 dropped out in subsequent intervals through 24 months after starting ART.

**Table 1:** Demographic characteristics of patient population at ART initiation

Patient characteristic at ART initiation	Descriptive statistic
Males, *n* (%)	9190 (36.4%)
Age in years, median (IQR)	38 (32–44)
CD4 cell count (cells/µL)^a^, median (IQR)	116 (53–180)
Year of ART initiation, *n* (%)	
2004	2141 (8.5)
2005	5725 (22.7)
2006	8650 (34.2)
2007	8458 (33.5)
2008	287 (1.1)

a CD4 count obtained within six months prior and up to 2 weeks after ART initiation.

Rates of discontinuation from follow-up (loss to follow-up), treatment discontinuation and observed mortality (among patients on observation) are presented in Table 2. Of note is the small percentage of patients in care who are off treatment, which remained between 1.5 and 2.1%. This is the motivating factor for carrying out a sensitivity analysis (see below).

**Table 2:** Loss to follow-up, observed mortality, and treatment access over time

Months after ART start	Loss to follow-up,* N (%)	Observed mortality,* N (%)	Off treatment,* N (%)
Baseline (0–3)	2929 (11.6)	1015 (4.0)	0 (0.0)
(3–9)	1502 (7.0)	403 (2.0)	417 (2.1)
(9–15)	1040 (5.4)	159 (0.9)	384 (2.1)
(15–21)	701 (3.8)	106 (0.6)	331 (1.9)
(21–27)	473 (2.7)	39 (0.2)	252 (1.5)

* Out of all patients under study at each time interval.

### Scenario 1: Had all patients remained on observation (and possibly on treatment)

**Figure 1:**
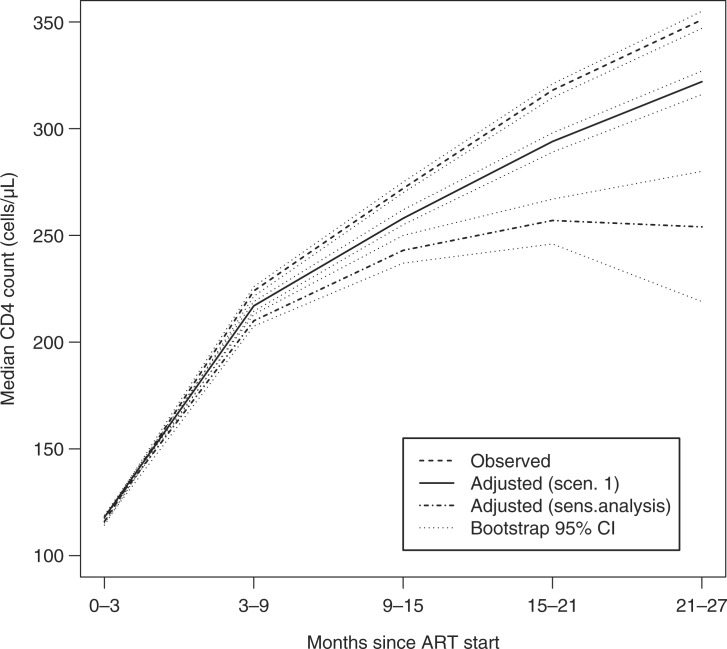
Overall non-weighted (dashed line) and IPCW-adjusted median CD4 count had all patients remained in care assuming equal survival and access to care (Scenario 1 solid line) and assuming 50% lower access to treatment and 80% survival compared to patients remaining in care (sensitivity analysis; heavy dash). Bootstrap-generated 95% confidence intervals are included (light dash). Note that, under the sensitivity analysis, this is a slightly modified Scenario 1, where the question centres simply on the patient being on observation (i.e. on solely knowing the outcome of the patients but not assuming that they have remained in care, as it is the case when no sensitivity analysis is performed).

Table 3 and Figure 1 show the non-weighted and IPCW-adjusted median CD4 cell counts over time. The IPCW-adjusted median CD4 count is up to 30 cells/µL lower than the estimate based on available data up to two years after the start of therapy (Figure 1). It should be noted that these analyses assume that mortality and treatment access patterns are identical among patients who remain in care and those who discontinue follow-up with the same observed measured characteristics (MAR).

**Table 3:** Non-adjusted and IPCW-adjusted median CD4 cell counts over time according to two counterfactual scenarios

Months after ART start	Median CD4 count (cells/µL)		

	Unadjusted	IPCW-adjusted	

	Median (IQR)	Scenario 1 Median (IQR)	Scenario 2 Median (IQR)
Baseline (0–3)	116 (53, 180)	118 (56, 180)	118 (56, 180)
(3–9)	224 (140, 333)	217 (131, 326)	217 (131, 326)
(9–15)	272 (177, 394)	258 (156, 381)	259 (158, 382)
(15–21)	318 (210, 452)	294 (179, 426)	297 (182, 329)
(21–27)	351 (238, 490)	322 (196, 459)	326 (202, 463)

### Scenario 2: Had all patients remained on observation and on treatment

**Figure 2:**
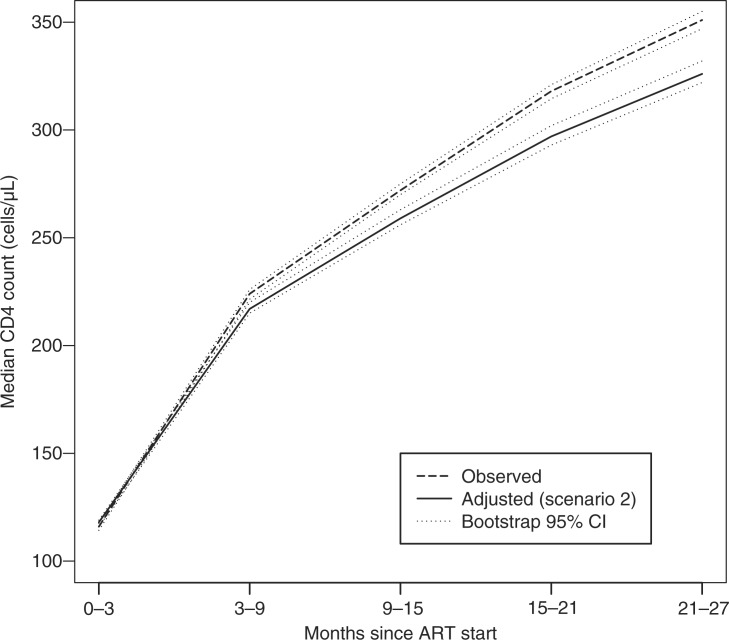
Overall non-weighted (dashed line) and IPCW-adjusted median CD4 count had all patients remained in care and on treatment assuming equal survival and access to care (Scenario 2 solid line).

The second question in this manuscript considers the median CD4 count over time had all patients remained on observation *and* continued treatment. The IPCW analysis showed that the estimated median CD4 count under Scenario 2 was slightly higher than the estimated median CD4 count under Scenario 1 during later periods, but this difference was not particularly pronounced (Figure 2).

### Sensitivity analyses

In the sensitivity analysis, we varied the survival and treatment access rates among the dropouts in order to investigate their effect on IPCW-adjusted CD4 counts. We assumed that survival and access to treatment would be lower among dropouts compared to patients in care with similar characteristics. The proportions were varied by multiplying the probability of remaining on treatment, as estimated among patients under observation, by a factor of 20, 50 and 80% and, similarly, the proportion of patients surviving, estimated among patients under observation, by a factor of 50 and 80%. In the case of treatment access, these factors represent published reports involving interviews among patients who discontinue from care and are subsequently found alive in the community [20], while the higher probability of death in patients who discontinue care has been extensively documented [9, 11, 24–26]. For brevity, we present here the sensitivity analysis under Scenario 1 corresponding to 50% treatment access and 80% survival among the dropouts, compared to similar patients under observation (Figure 1). Of note is the higher variability of the sensitivity-derived estimates reflected by the wider 95% bootstrap intervals. This increased variability reflects the underlying variability of the higher mortality rate and the lower access to ART among patients lost to clinic (binomial probabilities with higher variability) compared to patients on observation who experience very low mortality rates and extremely high access to care (binomial probabilities with very low variability).

### CD4 count estimates without incorporating mortality

We also analyzed longitudinal CD4 counts without adjusting for death (i.e. by not including any CD4 count values of −1 after death in the calculation of median CD4 counts at each interval). Supplementary Figure 1 shows longitudinal CD4 count estimates excluding data on patients who have died. These analyses used the sensitivity analytical procedures described earlier where it was assumed that, patients out of care have 50% lower rates of access to treatment compared to patients in care. Two estimates were produced: One assumed that survival was identical among patients under care (albeit accessing treatment at lower levels compared to patients in care) and one assumed that survival rates among patients who have discontinued from care at the original clinic were 20% lower than those continuing in care.

### Extensions

**Figure 3:**
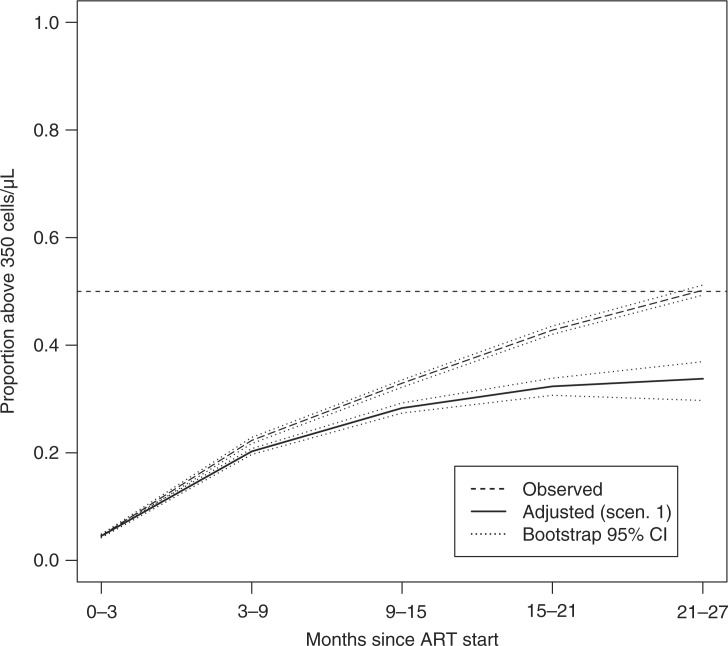
Extensions of the estimating procedure. Estimated proportion of patients alive with CD4 count > 350 cells/µL according to observed counts obtained solely from patients under observation (dashed line) and IPCW-adjusted estimates under Scenario 1 (solid line). The reference line is the 50% threshold. Bootstrap-generated 95% confidence intervals are included (light dash).

An additional summary measure involving longitudinal CD4 counts estimated under Scenario 1 is the proportion over time of patients with CD4 counts above 350 cells/µL. Defined in the same manner as the survival-adjusted median described above, this analysis addresses the question of what proportion of patients from the original cohort are alive with CD4 above 350 cells/µL after starting ART. Figure 3 presents the results of these analyses. Of note, we used the same estimates of treatment access and mortality among dropouts as presented in the sensitivity analysis section above (i.e. 80% survival and 50% rate of treatment access among dropouts compared to similar patients under observation), because we consider these as the most likely state of affairs. It is clear from the figure that, simply averaging data from patients who are alive and on observation, just over 50% will have CD4 counts above 350 cells/µL at two years from the start of therapy. The IPCW-adjusted estimate is 29%. The interpretation is that less than 30% of patients starting treatment will be alive with CD4 counts above 350 cells/µL two years later.

A final by-product of this analysis is a revised estimate of the mortality rates adjusting for differential dropout and higher mortality and lower treatment access rates among patients no longer in follow-up. Supplementary Figure 2 shows the routine cumulative mortality estimates produced by considering only observed deaths versus the adjusted estimates which consider both the characteristics of lost patients as well as an estimate of the mortality rate among patients no longer on observation. Assuming 20% lower survival among patients no longer in follow-up, the adjusted cumulative mortality rate is twice as high as the observed mortality rate at one year and more than three times at two years.

## Discussion

This study addressed two questions related to the estimation of CD4 counts over time after initiation of ART: “What would the median CD4 count be had all patients remained in care and possibly on treatment?” (Scenario 1) and “What would the median CD4 count be had all patients remained both in care and on treatment?” (Scenario 2). These are two important questions in the present context of rapid scale-up of ART in resource-limited settings. Scenario 1 addresses issues related to the entire cohort of patients starting therapy, including both those who continue treatment as well as those who stop or die. In generalized epidemics, such as the one in sub-Saharan Africa, Scenario 1 addresses, the global outcome of an entire community initiating treatment. Scenario 2 addresses the critical question of what benefit would have been derived for the patients if higher proportions of them had been maintained on treatment. The answer to this question provides important input in policy and decision-making particularly in the setting of mutually exclusive decisions on resource allocation (e.g. investing in retention of patients already under treatment or starting new patients on therapy) as well as choice of treatment regimens and substitution of more toxic with less toxic (but possibly more expensive) drugs.

An additional feature of this method is that it incorporates patient survival in the estimation of longitudinal CD4 counts. The interpretation of the median CD4 count over time, in the present analysis, is the CD4 count threshold in each interval where 50% of the *original population* of patients starting ART is alive with CD4 count above that threshold. Thus, in addition to measuring the effectiveness of a program in maximizing patient CD4 count response to treatment, program evaluation takes into account the program's success in keeping its clients alive, the ultimate criterion of program effectiveness.

Regardless of the approach, this study found a substantial positive bias in the estimation of CD4 counts when observations are obtained by simply averaging data from patients under observation. In fact, the difference between the routinely reported CD4 count and the adjusted estimate, which accounts for selective dropout and mortality by the sicker patients, diverged over time. This suggests that the subgroup of patients who remain in care are increasingly unrepresentative of the cohort initiating ART.

The answer to the counterfactual question in Scenario 2 (that is, what would be the estimated median CD4 count over time had all patients remained in care and on treatment), was similar to the one in Scenario 1 (had everyone remained in care; Figure 1). Given that the IPCW-adjusted estimate under Scenario 1 assumes that mortality and treatment access among dropouts is the same as among patients on observation, the lack of discernible differences in the estimates produced by the two scenarios is not surprising, since the majority of patients on observation received ART (Table 2). Thus, the results presented in Figure 1 assume correspondingly high rates of survival and access to treatment among dropouts as observed among similar patients under observation. More relevant is the comparison between the IPCW-adjusted estimates of CD4 counts over time assuming that a much lower proportion of patients who discontinue from care survive and are accessing ART compared to patients in care with similar characteristics. Comparing the trajectory associated with the sensitivity analysis under Scenario 1 shown in Figure 1 to the trajectory under Scenario 2 shown in Figure 2, provides clear evidence of the advantages conferred on patients who remain both in care and on treatment. The median CD4 count over time estimated under Scenario 2 is much higher than the adjusted median CD4 count under Scenario 1 which assumes lower rates of treatment access and higher mortality among patients who have discontinued from observation. The conclusion from the results presented in Figures 1 and 2 is that, under realistic projections of mortality and treatment access, the resulting CD4 trajectory is much lower than the CD4 trajectory obtained from averaging data from patients who continue participating in care. By contrast, the trajectory estimated under Scenario 2 (i.e. had everyone remained both on observation and on treatment) results in much higher CD4 counts compared to the trajectory under Scenario 1.

It is notable that, despite the robust benefit associated with maintaining patients on treatment, these estimates, produced under the best-case scenario of maintaining the entire patient cohort on treatment once ART has been initiated, were still lower than the unadjusted estimates, which are based on averaging data from patients in care (Figure 2). This shows that the usually reported estimates of CD4 count are significantly upwardly biased; or that they are simply addressing the much more narrow and possibly less relevant question, “What is the CD4 count over time *given that patients remain alive and in care*?” This question is irrelevant from the patient-level perspective because it is not known at the start of treatment which patients will remain in care and/or survive in the future. It is also of dubious usefulness at the population level as it is obtained from a cohort which bears decreasing resemblance with the one starting therapy, particularly as the time from ART initiation increases.

We found that, considering data solely on patients under observation, about 50% of patients are estimated to have CD4 counts > 350 cells/µL two years after ART starts. This is potentially a remarkable achievement of treatment with ART given that, at the start of therapy, the median CD4 count in the cohort initiating therapy was barely above 100 cells/µL (Table 1). However, the IPCW-adjusted estimates seem to tell a different story. According to this approach, and assuming realistic rates of mortality and access to treatment among dropouts, just over 30% of patients will be expected to alive with CD4 counts above 350 cells/µL after two years of therapy.

We have also carried out an analysis without incorporating death in the estimation of CD4 count (Supplementary Figure 1). CD4 count estimates in patients who were alive at each interval of estimation show that a similar, albeit attenuated, downward adjustment of the routine estimates results when taking into account differential dropout rates among patients.

A final by-product of our analytical approach is that our analysis produced adjusted mortality rates. This is because any measure amenable to averaging can be assessed with this methodology. (A corollary concerns analyses of adherence measures, which can be heavily biased when only considering data from patients who are alive and still in care and not accounting for those who have stopped therapy or are deceased; two outcomes strongly associated with lack of adherence. This however is the objective of a future research article.) The IPCW methodology produces adjusted mortality estimates which incorporate differential patterns of dropout and higher rates of mortality among patients who discontinue care. As shown in Supplementary Figure 2, assuming 25% higher mortality (20% lower survival) among patients who are lost to clinic, results in almost double one-year and two-year mortality rates as compared to the usual estimates based on observed data. Differences are even more pronounced if higher mortality is assumed among dropouts. Under the best-case scenario where all patients are maintained in care and on treatment (Scenario 2), mortality is substantially lower than all the estimates generated under Scenario 1 but still somewhat higher than the routine mortality estimates. This is consistent with the overestimation of CD4 counts under routine estimation even compared to the best-case Scenario 2 and shows how much mortality is potentially underestimated using routine methods. Finally, note that the one-year mortality rate of about 11% reported in Yiannoutsos *et al*. in the same cohort [11] corresponds more closely with the curve associated with 20% lower survival and 50% lower access to care among patients no longer on observation.

A major limitation of this study is that, due to lack of additional data on CD4 counts past discontinuation from care, we modelled the CD4 trajectories of patients out of care and treatment, by CD4 counts of patients not on treatment but still in care. It is questionable whether this is appropriate. Remaining in care may be advantageous, even if treatment has been interrupted, because of access to medical care and prophylaxis treatment (e.g. access to co-trimoxazole prophylaxis) as well as other ancillary services. Thus, remaining in care will be likely associated with a less steep decline in CD4 count and less sharp increases in morbidity and mortality rates compared to patients who are out of care. For this reason, even the significantly downwardly adjusted estimates of the median CD4 count after ART initiation should be considered as an upper bound of the true median CD4 count (in other words, median CD4 counts over time may be lower still).

An additional limitation of this analysis is that we have no information on connection to care and survival status among patients no longer on observation. That is, we do not know how many of these individuals continue receiving care and treatment services at other facilities. Recent work has shown that, particularly in regions with robust scale-up of care and treatment services, an increasing proportion of patients who are lost to follow-up have simply moved to another clinic providing ART [22, 27]. We have performed sensitivity analyses to assess the impact of various levels of self-referral on our estimates. The results are consistent with expectation. That is, the downward adjustment is more pronounced under scenarios where increasingly higher fractions of patients lost to clinic are not connected with another care and treatment program (analysis not shown). This suggests that this information should be collected as part of routine program evaluation at least in a subset of the dropout population [20, 27]. Similarly, sensitivity analyses assuming increasingly high mortality rates among dropouts followed predictable trends particularly when combined with increasing rates of disengagement from care. Additional information on the outcome of patients after loss to program (i.e. mortality under treatment and/or treatment access elsewhere) as well as the theory addressing the use of these auxiliary data is the objective of a future publication.

This study indicates that there is significant overestimation of the CD4 response to treatment for a cohort starting ART when available data are simply averaged to estimate long-term CD4 response to therapy. At the same time, retention of patients to treatment results in a salutary effect which manifests in much higher median CD4 counts over time. Policies, programs and ART regimens which improve patient retention into care and treatment are desirable and will significantly improve patient outcomes. IPCW is an elegant and intuitive methodology, which can be generalized to other settings and longitudinal measures (e.g. treatment adherence levels, HIV RNA–viral load measurements) and can be implemented using existing statistical software to address a number of critical clinical and public-health questions. With these methods rational decisions of the likely outcome of a number of interventions can be made based on more accurate quantification of the likely impact of patient behaviour, access to treatment and patient retention efforts.

## Appendix 1

We present here an outline of the statistical procedures involved in the sensitivity analyses. These involve relaxing the reliance on the missing-at-random (MAR) assumption to calculate the inverse probability of not being censored by the last visit *k* given the complete covariate ${\bar{L}}_{K}$ and treatment history ${\bar{A}}_{K}$ up to *K*, that is, $P\left(C_{K}=0|{\bar{L}}_{K},{\bar{A}}_{K}\right)$ involved in the IPCW weights. Under MAR, this probability can be analyzed into products of probabilities, based on data up to each visit *k*=1, … ,*K* that is,$P(C_{K}=0|{\bar{L}}_{K},{\bar{A}}_{K})=\prod_{k=1}^{K}P(C_{k}=0|{\bar{L}}_{k-1},{\bar{A}}_{k-1},C_{k-1}=0)$

### Scenario 1

Depending on the type of scenario these probabilities can be further simplified. For example, in patients who have died between visits *k*-1and *k*, we can relax the MAR assumption by considering that

$$P(C_{k}=0|{\bar{L}}_{k-1},{\bar{A}}_{k-1},C_{k-1}=0)=\frac{P(C_{k}=0,D_{k}=1|{\bar{L}}_{k-1},{\bar{A}}_{k-1},C_{k-1}=0)}{P(D_{k}=1,|{\bar{L}}_{k-1},{\bar{A}}_{k-1},C_{k-1}=0)}$$

The numerator can be estimated through the usual means (routine application of the Bayes theorem) but the denominator cannot be estimated without reliance on MAR, in other words, relying on the assumption that the probability of mortality is the same in patients lost to clinic at visit *k* (i.e.C_*k*_=1), as those on observation (C_*k*_=0). More specifically, the MAR assumption guarantees that

$$P(D_{k}=1|C_{k}=1,{\bar{L}}_{k},{\bar{A}}_{k},C_{k-1}=0)=P(D_{k}=1|{\bar{L}}_{k-1},{\bar{A}}_{k-1},C_{k}=0)$$

If vital status data are available on a sample of patients lost to clinic [11, 24] the probability $P(D_{k}=1|C_{k}=1,{\bar{L}}_{k-1},{\bar{A}}_{k-1},C_{k-1}=0)$ can be modelled explicitly. In the current analysis we have assumed that

$$1-P(D_{k}=1|C_{k}=1,{\bar{L}}_{k-1},{\bar{A}}_{k-1},C_{k-1}=0)=0.8*(1-P(D_{k}=1|{\bar{L}}_{k-1},{\bar{A}}_{k-1},C_{k-1}=0))$$

Or, in other words, that the survival rates of patients lost to clinic are, on average, 80% of those still in care.

Also, for patients alive and on ART at *k* the above probability involves factors such as

$$P(C_{k}=0|{\bar{L}}_{k},{\bar{A}}_{k},C_{k-1}=0)=\frac{P(C_{k}=0,D_{k}=0,A_{k}=1|{\bar{L}}_{k-1},{\bar{A}}_{k-1},C_{k-1}=0)}{P(D_{k}=0,A_{k}=1|{\bar{L}}_{k-1},{\bar{A}}_{k-1},C_{k-1}=0)}$$

The MAR assumption specifies that access to ART among patients who are alive is the same among those lost to clinic as among those who remain under observation with the same characteristics at the time of dropout, that is

$$P(D_{k}=0,A_{k}=1|C_{k}=1,{\bar{L}}_{k-1},{\bar{A}}_{k-1},C_{k-1}=0)=P(D_{k}=0,A_{k}=1|C_{k}=0,{\bar{L}}_{k-1},{\bar{A}}_{k-1})$$

In the forthcoming methodological publication we use data from a sample of the dropouts to model the probability $P(D_{k}=0,A_{k}=1|C_{k}=1,{\bar{L}}_{k-1},{\bar{A}}_{k-1},C_{k-1}=0)$, but in the current publication we used sensitivity analyses, where we assumed that

$$P(A_{k}=1|D_{k}=0,C_{k}=1,{\bar{L}}_{k-1},{\bar{A}}_{k-1},C_{k-1}=0)=0.5P(A_{k}=1|D_{k}=0,C_{k}=0,{\bar{L}}_{k-1},{\bar{A}}_{k-1})$$

(i.e. that the access to care among live patients lost to clinic is half of that among patients still on observation. Note that the opposite scenario, of alive patients off treatment, we set

$$P(A_{k}=0|D_{k}=0,C_{k}=1,{\bar{L}}_{k-1},{\bar{A}}_{k-1},C_{k-1}=0)=1-P(A_{k}=1|D_{k}=0,C_{k}=1,{\bar{L}}_{k-1},{\bar{A}}_{k-1}C_{k-1}=0)$$

### Scenario 2

For scenario 2, matters are simpler, as patients who stop treatment are censored. Thus, only the probability of death among patients lost to care is relevant (with the additional requirement that a patient remained on ART up to the moment of loss to clinic). We have made the same assumptions in the sensitivity analysis as before (i.e. that the survival among patients lost to clinic is 80% of the respective survival of those still on observation).
